# Exploring native genetic elements as plug-in tools for synthetic biology in the cyanobacterium *Synechocystis* sp. PCC 6803

**DOI:** 10.1186/s12934-018-0897-8

**Published:** 2018-03-26

**Authors:** Deng Liu, Himadri B. Pakrasi

**Affiliations:** Department of Biology, Washington University, Campus Box 1137, One Brookings Drive, St. Louis, MO 63130 USA

**Keywords:** Cyanobacteria, Promoters, RBS, Terminators, Plasmids

## Abstract

**Background:**

The unicellular cyanobacterium *Synechocystis* sp. PCC 6803 has been widely used as a photoautotrophic host for synthetic biology studies. However, as a green chassis to capture CO_2_ for biotechnological applications, the genetic toolbox for *Synechocystis* 6803 is still a limited factor.

**Results:**

We systematically characterized endogenous genetic elements of *Synechocystis* 6803, including promoters, ribosome binding sites, transcription terminators, and plasmids. Expression from twelve native promoters was compared by measuring fluorescence from the reporter protein EYFP in an identical setup, exhibiting an 8000-fold range of promoter activities. Moreover, we measured the strength of twenty native ribosome binding sites and eight native terminators, indicating their influence on the expression of the reporter genes. In addition, two shuttle vectors, pCA-UC118 and pCB-SC101, capable of replication in both *Synechocystis* 6803 and *E. coli* were constructed. Expression of reporter proteins were significantly enhanced in cells containing these new plasmids, thus providing superior gene expression platforms in this cyanobacterium.

**Conclusions:**

The results of this study provide useful and well characterized native tools for bioengineering work in the model cyanobacterium *Synechocystis* 6803.

**Electronic supplementary material:**

The online version of this article (10.1186/s12934-018-0897-8) contains supplementary material, which is available to authorized users.

## Background

Cyanobacteria are the only prokaryotic species capable of oxygenic photosynthesis and have been attractive as photoautotrophic factories to convert CO_2_ and H_2_O into valuable products [1, 2]. As the first cyanobacterium with a sequenced genome [3], *Synechocystis* sp. PCC 6803 (here after *Synechocystis* 6803) has been widely used as a host for metabolic engineering and synthetic biology studies [4]. However, when compared to *Escherichia coli* (*E*. *coli*), the genetic toolbox for bioengineering work in *Synechocystis* 6803 is not optimal, especially when multiple genes in multiple operons need to be manipulated [5].

Promoters are the genetic elements that are the best characterized to date in *Synechocystis* 6803. Many native promoters have been characterized, including the strong promoters P_*psbA2*_, P_*rbcL*_, P_*cpcB*_, and their derivatives [6–8], as well as the metal inducible promoters P_*nrsB*_, P_*coaT*_, P_*petE*_, and P_*ziaA*_ [9, 10], and the light-control promoter P_*cpcG2*_ [11]. The well-known promoters from *E. coli* such as P_*tetR*_, P_*lacO*_, P_*trc*_, and their derivatives have also been characterized in *Synechocystis* 6803. However, these come with significant challenges such as light sensitivity of the inducer anhydrotetracycline for P_*tetR*_, and not ideally working as in *E. coli* for the *lacI*-type promoters [12, 13].

Ribosome binding sites (RBS) are effective control elements for translation initiation. A library of expression elements with various strengths of RBS is useful for tuning protein expression levels when multiple genes are organized into one operon. Unlike promoters, only a few RBS have been characterized in *Synechocystis* 6803, such as the RBS in the *psbA2* and *rbcL* genes [9], several RBS from BioBrick Registry of standard biological parts (http://parts.igem.org/) as well as a synthetic one named RBSv4 and its derivatives [8].

Transcription terminators are additional important genetic elements to ensure that expression of any engineered gene does not affect the transcription of any downstream gene. It is believed that only one transcriptional termination mechanism exists in *Synechocystis* 6803, namely Rho-independent termination [14], because genes coding for homologues of *E. coli* Rho proteins have not been found in the *Synechocystis* 6803 genome. Rho-independent terminators are composed by a loop motif within the transcript, a GC-rich RNA hairpin structure followed by a U-rich tail sequence, both of which are necessary for termination [15]. The endogenous Rubisco terminator T_*rbcS*_ and the *E. coli* terminator T_*rrnB*_ are the only two terminators presently used in bioengineering studies with *Synechocystis* 6803 [16, 17]. Since reuse of genetic elements in a genetic design might lead to homologues recombination [18], additional terminators need to be explored in order to express genes in multiple operons in *Synechocystis* 6803. This is important since it has been shown that the fragment between two identical sequences could be discarded in *Synechocystis* 6803 duo to genetic recombination [19].

Integrating target genes to neutral sites on the chromosome through double homologous recombination has been the strategy preferentially used for decades for genetic manipulation of *Synechocystis* 6803 [20]. A number of neutral sites have been identified for gene expression in this cyanobacterium [21]. This strategy allows stable expression of introduced genes in cyanobacterial cells, but final genome segregation is a time-consuming process. More importantly, the length of the integrating part is a limiting factor for homologous recombination.

There are multiple choices of plasmids that can be used as gene expression platforms in *E. coli*. However, only the pRSF1010-based plasmid, a broad-host-range vector, has been used as a platform for gene expression in *Synechocystis* 6803 [22]. Having only one plasmid for gene expression is a limiting factor for synthetic biology work in this cyanobacterium.

The purpose of this study is to develop native genetic elements for convenient plug-in use in *Synechocystis* 6803. To enrich the promoter toolbox, nine additional native promoters were characterized for their expression characteristics. In total, thirteen promoters including P_*psbA2*_, P_*rbcL*_, P_*cpcB*_, and P_*trc1O*_, were compared for their strengths under standardized conditions. For the RBS library, twenty RBS elements were compared on their strengths for translation initiation under the same promoter. We also established a small library of terminators, each with different strengths to stop transcription. In addition, two shuttle vectors were constructed, which provide additional, new platforms for genes expression in *Synechocystis* 6803 beyond the known single expression vector.

## Results and discussion

### Comparison of the activities of native promoters

**Fig. 1:**
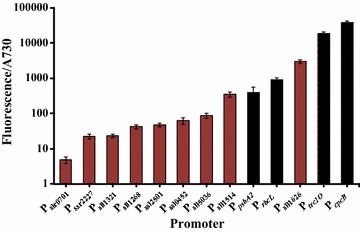
Promoter activities in *Synechocystis* 6803. Activities were measured as EYFP fluorescence per Abs_730_ for cell cultures in BG11 medium. New promoters characterized in this study are shown with red bars. Error bars indicate standard deviations (*n *= 3)

In order to compare the strengths of native promoters in *Synechocystis* 6803, 5′-UTR sequence before the translational start codon of each gene was amplified by PCR as an intact promoter, which was then directly placed before the gene coding an enhanced yellow fluorescence protein (EYFP) as a reporter protein [23]. Since the 5′-UTR sequence of the corresponding gene contains both the promoter region and RBS, we have used ‘PR’ to represent such a 5′-UTR. Following the reporter gene, terminator T_*rrnB*_ was used to form the expression cassette “PR_testing_ + eyfp + T_*rrnB*_”, which was cloned in the replicating plasmid pRSF1010. The native promoters PR_*psbA2*_, PR_*rbcL*_, PR_*cpcB*_, and the *E. coli* originated promoter PR_*trc1O*_ have been characterized previously [6, 9, 17], and all of them exhibit strong abilities to drive transcription. Besides these four promoters, we chose additional nine native *Synechocystis* 6803 promoters (shown in Fig. 1 and Additional file 1: Table S1) to be compared simultaneously for the activity. All of these nine 5′-UTR have their own unique “-35” regions, “-10” regions, and transcription start sites (TSS) based on the published RNA-seq data for *Synechocystis* 6803 [24]. The sequence length of each 5′-UTR was selected based on their own genomic context. Briefly, the sequence between the start codon of the corresponding gene and the stop codon or the 5′-UTR of the upstream neighboring gene was used to define the length of each native 5′-UTR fragment. The functions of corresponding genes of all native promoters described in this study are shown in Additional file 1: Table S2.

By analyzing fluorescence intensities for these thirteen promoters (Fig. 1), we found that the strongest expression was from PR_*cpcB*_, having the identical sequence of the promoter defined previously as a super strong-promoter P_cpc560_ [25]. Compared to PR_*cpcB*_, the weakest promoter element PR_slr0701_ showed about 8000-fold less EYFP fluorescence intensity, which indicated the broad range of the strengths of promoters studied here. The promoter PR_sll1514_ showed a similar activity to that of PR_*psbA2*_. We identified another strong promoter PR_sll1626_, having the strength between PR_*rbcL*_ and PR_*trc1O*_. This study differs from previous work, which modified nucleotides within a promoter to change the strengths of expression [6, 8]. We used various native 5′-UTR with different sequences to prevent genetic recombination. Although native promoters characterized in this part are the sequences of 5′-UTR of corresponding genes, containing both the promoter region and RBS, we treated them as intact elements for plug-in use for engineered gene expression in *Synechocystis* 6803. Our results suggest that unexplored native promoters constitute an important resource for cyanobacteria for application in synthetic biology.

### Characterization of the 22-bp native RBS

**Fig. 2:**
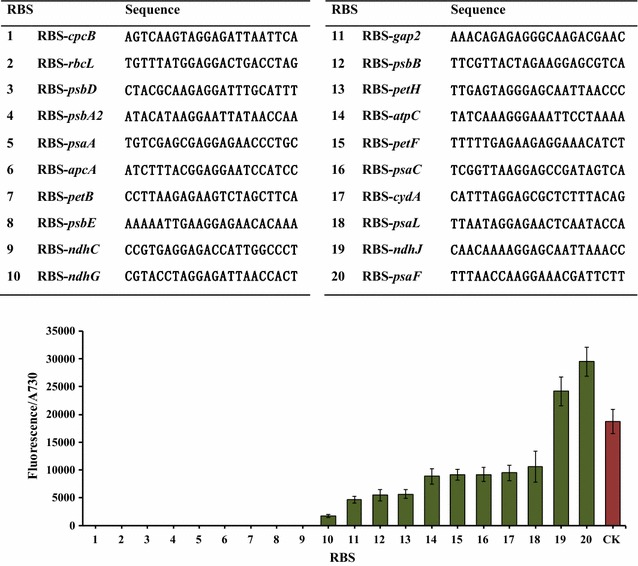
Comparison of strengths of ribosome binding sites. All constructs were driven by P_*trc1O*_ with variations of the 22-bp fragment before the start codon of the *eyfp* gene. Activities were measured as EYFP fluorescence per Abs_730_ for cell cultures in BG11 medium. CK represents the plasmid with original 22-bp sequence of P_*trc1O*_ promoter. Error bars indicate standard deviations (*n *= 3)

To setup the RBS library composed of standard elements, all twenty native RBS sequences studied here were exactly 22-bp upstream of the translational start codon of the respective genes, most of them coding for photosystem related proteins (Fig. 2). All of the 22-bp native RBSs studied here have the typical “AG” rich regions before the translational start codon. For the purpose of constructing an operon with multiple genes, an overlap with 22-bp RBS sequence works efficiently in the Gibson Assembly method used to generate the expression constructs [26]. To compare the strength of RBS sequences, P_*trc1O*_ was selected as the promoter to drive transcription of the reporter gene. In the experiments, the last 22 nucleotides of PR_*trc1O*_ were replaced by one of the twenty native RBS sequences, followed by the gene coding for the EYFP protein. The terminator T_*rrnB*_ was added behind the *eyfp* gene to form the reporter cassette “P_*trc1O*_+ RBS-testing + *eyfp *+ T_*rrnB*_”, before placing into the pRSF1010 vector.

RBS-*ndhJ* and RBS-*psaF* showed higher activities for translation initiation than the other eighteen RBS elements, as well as that of the control RBS of P_*trc1O*_ (Fig. 2). Interestingly, we could not detect the fluorescence signals from nine of these strains expressing EYFP, including the RBS-*cpcB*, RBS-*rbcL*, and RBS-*psbA2*, which all have activities with their own promoter as shown above (Fig. 1). We checked the nine strains by PCR to verify that each of the plasmids with the testing cassette was intact in cyanobacterial cells (Additional file 1: Figure S1). It has been reported that the activities of RBS can vary in a broad range depending on the sequence context [27]. Similar results have been previously reported in *Synechocystis* 6803 [8, 9]. Prediction of an effective length of RBS as tools for genetic engineering work is challenging, and in this study we used RBS sequences of identical lengths as standard elements for genetic manipulation. Although activities of RBS elements determined here were relative strengths based on the EYFP protein as a reporter, we have continued to develop an RBS library with permutations of different standard elements, which will be useful in future for manipulation of multiple genes within operons for expression in *Synechocystis* 6803.

### Establishment of a transcription terminator library

**Fig. 3:**
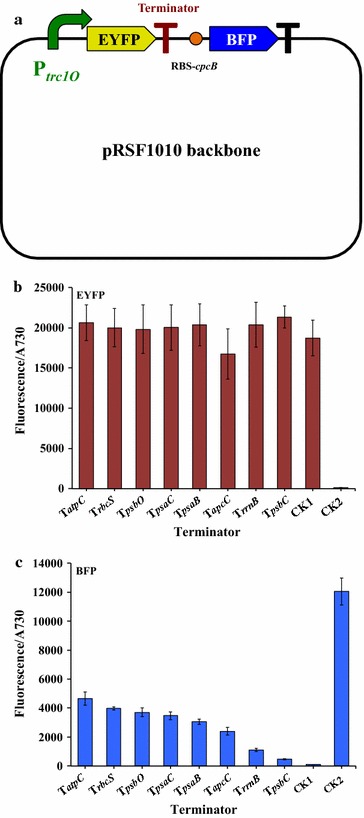
Measurement of strengths of transcription terminators. **a** Terminators were inserted between genes for two fluorescence proteins in the plasmid. RBS, ribosome binding sites. **b**, **c** Fluorescence from protein EYFP and BFP were measured, respectively, as fluorescence per Abs_730_ for cell cultures in BG11 medium. CK1 represents the plasmid only with the *eyfp* gene, while CK2 represents the plasmid only with the *bfp* gene. Error bars indicate standard deviations (*n *= 3)

A terminator library is currently unavailable for *Synechocystis* 6803. To determine terminator characteristics, we undertook a strategy previously used for *E. coli* to test the strengths of native terminators in *Synechocystis* 6803 [28]. Driven by PR_*trc1O*_ promoter, the *eyfp* gene preceded different individual terminators in the reporter construct. We added an internal control by placing a second complete reporter cassette as follows. Behind the initial testing terminator cassette, the RBS-cpcB was added 5′ to the second reporter gene, coding for blue fluorescence protein (BFP) [29], followed by another terminator T_*rrnB*_ (or T_*rbcS*_ when testing terminator is T_*rrnB*_). The entire double cassette “PR_*trc1O*_+ eyfp + T_testing_ + RBS-*cpcB *+ *bfp *+ T_*rrnB*_” was ligated to the plasmid pRSF1010 (Fig. 3a). We reasoned that the stronger terminators in the library would lead to less fluorescence intensity from protein BFP. Additional two plasmids were constructed as controls, CK1 and CK2, containing the expression cassettes “PR_*trc1O*_+ *eyfp *+ T_*rrnB*_” and “P_*trc1O*_+ RBS-*cpcB *+ *bfp *+ T_*rrnB*_”, respectively.

Besides the widely used but non-native terminator T_*rrnB*_, we compared the strengths of seven native terminators, of which the corresponding genes coding for photosynthesis related proteins. All of these terminators have a typical structure of Rho-independent termination (labeled sequences in Additional file 1: Table S2). The fluorescence intensity from EYFP was the same for all eight strains with different terminators compare to the strain containing the plasmid CK1 (Fig. 3b). However, the intensity from BFP showed a tenfold difference between T_*atpC*_ and T_*psbC*_ (Fig. 3c). In fact, none of the terminators tested in this study can completely stop the transcription driven by the PR_*trc1O*_ promoter. These results testing a small library of terminators indicate that terminators are important factors that need to be considered in future bioengineering work in *Synechocystis* 6803.

Interestingly, we could detect the BFP fluorescence from the strain containing the plasmid CK2, containing the expression cassette “P_*trc1O*_+ RBS-*cpcB *+ *bfp *+ T_*rrnB*_”. However, we could not detect the fluorescence using the same cassette but using *eyfp* as the reporter gene, “P_*trc1O*_+ RBS-*cpcB *+ *eyfp *+ T_*rrnB*_” (Fig. 2). On the other hand, when we used the promoter P_*cpcB*_ to form the cassette “PR_*cpcB*_+ *eyfp *+ T_*rrnB*_”, EYFP protein was expressed as shown in Fig. 1. All three cassettes have the same RBS, but different sequence contexts (promoters and following genes), affecting protein expression. This showed that sequence context is an important factor to influence gene expression.

### Two shuttle vectors as gene expression platforms

As mentioned above, it is necessary to develop additional platforms to efficiently express genes on self-replicating plasmids in *Synechocystis* 6803 beyond using the pRSF1010 plasmid as the only exclusive platform. The necessity was promoted especially since CRISPR-Cpf1 system has been reported as a powerful and efficient tool to edit the genome of *Synechocystis* 6803 [30]. Components of the CRISPR system including the gene coding for the protein Cpf1 has been engineered into the plasmid pRSF1010, which is eventually cured for the purpose of completely markerless editing of the genome. If the CRISPR-Cpf1 system is utilized to edit genes in *Synechocystis* 6803, there is a need for additional shuttle vectors to express multiple genes or operons for complex pathways or bioprocesses, which might be very difficult to integrate into the chromosome because of their large size.

*Synechocystis* 6803 contains three small endogenous plasmids pCA2.4, pCB2.4 and pCC5.2. It has been shown that integration of interesting genes into pCA2.4 and pCC5.2 resulted a higher expression profile than into the chromosome or the replicating plasmid pRSF1010 [21, 31]. The higher expression is presumably caused by a higher copy number of the endogenous small plasmids within the cyanobacterial cells than the copy numbers for the chromosome or pRSF1010 [32]. Additionally, we determined that these three native plasmids could not replicate in *E. coli* (data not shown), which could be due to a lack of recognition of the specific origin of replication (*ori*). Since many plasmids are known to be able to replicate in *E. coli*, combining plasmid backbones of *E. coli* vectors with the endogenous plasmids of *Synechocystis* 6803 should generate shuttle vectors between *E. coli* and *Synechocystis* 6803. With the use of such shuttle vectors, DNA cloning work could be carried out in *E. coli*, with the subsequent expression and/or analysis of interesting genes or operons after transformation of the same vectors into *Synechocystis* 6803. A similar strategy to construct a shuttle vector has been used in another cyanobacterium *Synechococcus elongatus* PCC 7942, based on the endogenous plasmid pANS [33]. This shuttle vector however, does not replicate in *Synechocystis* 6803.

**Fig. 4:**
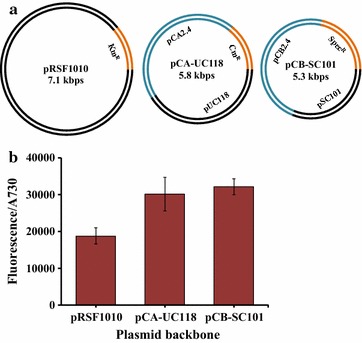
Replicating plasmids for expressing genes in *Synechocystis* 6803. **a** Schematic map of three replicating plasmids with different marker genes for antibiotic-resistance selection. K_m_^R^, C_m_^R^, and Spec^R^ represent the resistance genes for kanamycin, chloramphenicol, and spectinomycin, respectively. **b** Comparison of fluorescence from EYFP protein in three strains with different plasmids. The fluorescence data was normalized with Abs_730_ for cell cultures in BG11 medium with corresponding antibiotics. Error bars indicate standard deviations (*n *= 3)

To construct a shuttle vector of a smaller size than pRSF1010, we chose plasmids pCA2.4 and pCB2.4 in this study. The backbones of a high copy number plasmid pUC118 [34], and a low copy number plasmid pSC101 [35], were individually combined with pCA2.4 and pCB2.4, respectively. After the addition of antibiotics resistance genes, two shuttle vectors pCA-UC118 and pCB-SC101 were generated (Fig. 4a). To test whether they can replicate and serve as platforms for gene expression in *Synechocystis* 6803, the expression cassette “PR_*trc1O*_+ *eyfp *+ T_*rrnB*_” was placed into pCA-UC118 and pCB-SC101, respectively. The plasmid pRSF1010 was used as a control. These shuttle vectors were successfully transferred into *Synechocystis* 6803 by natural transformation [36]. *Synechocystis* 6803 cells containing either shuttle vector construct exhibited EYFP fluorescence intensities 50% higher than from cells containing pRSF1010 (Fig. 4b). The results demonstrated that pCA-UC118 and pCB-SC101 work efficiently as expressing platforms in *Synechocystis* 6803. In addition, we transferred the plasmids pCA-UC118 and pCB-SC101 sequentially into the strain containing the pRSF1010 plasmid with the expression cassette “PR_trc1O_ + *eyfp *+ T_*rrnB*_”. The strain containing three engineered plasmids grew in the medium with all three corresponding antibiotics, and maintenance of three plasmids in one host was verified by PCR (Additional file 1: Figure S2). This suggested that the compatibility is not an issue for all three plasmids as platforms to engineer gene expression in *Synechocystis* 6803.

## Conclusions

During this study, we investigated the strength of native genetic elements in *Synechocystis* 6803, including promoters, RBS, and terminators using a standardized genetic setting for comparisons. We also constructed two unique shuttle vectors for gene expression, resulting in four different sets of genetic manipulating platforms: the chromosome, pRSF1010, pCA-UC118, and pCB-SC101, that can be used in *Synechocystis* 6803. The libraries of each element provide a rich toolbox with multiple options for synthetic biology studies. It is notable that the relative activities assayed here varied depending on the genetic context. Based on the useful plug-in genetic elements with variant activities described here, we have significantly advanced the use of utilizing *Synechocystis* 6803 as an efficient autotrophic green factory for biotechnology applications.

## Methods

### Strains and culture conditions

All cloning was performed in *E. coli* strain XL1-Blue grown in LB medium in culture tubes or on agar plates at 37 °C, supplemented with 50 µg/ml kanamycin, 20 µg/ml chloramphenicol, or 30 µg/ml spectinomycin, as needed. *Synechocystis* sp. PCC 6803 cells were grown in BG11 medium [37] supplemented with 30 µg/ml kanamycin, 20 µg/ml chloramphenicol, or 20 µg/ml spectinomycin, as needed, under continuous white light at 30 µmol/m^2^/s at 30 °C. Cultures were grown in 125-ml glass flasks, in TPP tissue culture treated 6-well plates (Sigma-Aldrich), or on agar plates.

### Plasmids construction

All the plasmids used in this study are listed in Additional file 1: Table S1, which were constructed by the Gibson Assembly method [26], using linear fragments purified from PCR products. The promoter and terminator sequences from Additional file 1: Table S2 were amplified by PCR using *Synechocystis* 6803 genomic DNA as template. The RBS sequences were selected as 22-bp immediately preceding the translational start codon of each gene. The DNA fragments for construction of the plasmids pCA-UC118 and pCB-SC101 were amplified from *Synechocystis* 6803 genomic DNA, plasmid pUC118 [34], and plasmid pSC101 [35], respectively. All of the plasmids for assay of promoter, RBS, and terminator activities were ligated to the plasmid backbone pRSF1010, which is a derivative of the pPMQAK1 broad host range vector [6].

All PCR amplifications were performed using Phusion High-fidelity DNA polymerase (Thermo Scientific). Plasmids and PCR products were purified using the GeneJET (Thermo Scientific) plasmid miniprep kit and gel extraction kit, respectively. Oligonucleotides were designed using the SnapGene software (GSL Biotech LLC) and synthesized by IDT (Coralville, IA). All oligonucleotides used in this study are listed in Additional file 1: Table S3.

### Transformation of *Synechocystis* 6803

A tri-parental conjugation method was used to transfer all pRSF1010 derivative plasmids to *Synechocystis* 6803 wild-type cells, using a helper strain of *E. coli* containing the pRL443 and pRL623 plasmids [38]. For plasmids derivative from pCA-UC118 and pCB-SC101, *Synechocystis* 6803 cells were transformed with 500 ng(s) plasmids DNA via natural transformation [36]. Transformants were isolated on BG11 agar plates containing 20 µg/ml kanamycin, 10 µg/ml chloramphenicol, or 20 µg/ml spectinomycin, as needed. Isolated *Synechocystis* 6803 transformants was checked by PCR to confirm presence of the desired constructs.

### Fluorescence measurements

Each engineered strain with the desired plasmid was pre-cultured in 50 ml of BG11 medium with antibiotics in a 125-ml Erlenmeyer glass flask for 5 days. All cultures were adjusted to similar cell densities, with an OD_730 nm_ at 0.2 (about 1 × 10^8^ cells/ml) at the start of the experiment. Three independent replicates of each culture were then transferred to 6-well plates for 3 days of growth, followed by fluorescence measurements. The fluorescence intensity and the optical density of each culture were determined in 96-well black-walled clear-bottom plates on a BioTek Synergy Mx plate reader (BioTek, Winooski, VT). The excitation and emission wavelengths were set to 485 and 528 nm for EYFP, and 395 and 451 nm for BFP, respectively. All measured fluorescence data were normalized by culture density.

## Additional file

**Additional file 1.** Additional figures and tables.

## Abbreviations

EYFP: enhanced yellow fluorescence protein
RBS: ribosome binding sites
UTR: untranslated region
PR: promoter and RBS
TSS: transcriptional start sites
PCR: polymerase chain reaction
BFP: blue fluorescence protein
CRISPR: clustered regularly interspaced short palindromic repeats
