# Supplementary material for: Exploring native genetic elements as plug-in tools for synthetic biology in the cyanobacterium Synechocystis sp. PCC 6803
Source: Microb Cell Fact. 2018 Mar 26;17:48. doi: 10.1186/s12934-018-0897-8 (PMC5868059; doi:10.1186/s12934-018-0897-8)
Supplement: Supplementary file 1 — Additional file 1. Additional figures and tables. [file 12934_2018_897_MOESM1_ESM.docx]

**Figure S1** PCR analysis of plasmids with nine different RBS sequences (RBS-1 to RBS-9). Primers targeting the 5' and 3' regions of the “P*_trc1O_*+RBS-testing+*eyfp*+T*_rrnB_*” cassette on the plasmid were used for PCR.

**Figure S2** Presence of three replicating vectors in *Synechocystis* 6803. The *ori* regions were labeled in each plasmid. The origins for plasmids pCA2.4 and pCB2.4 are predicted. Black arrows on the plasmid maps indicate the primers used to check the presence of the plasmids in cells by PCR. In the gel, Lanes 1-3 show PCR results for the strain containing all three plasmids, lanes 4-6 show results for *Synechocystis* 6803 wild-type strain as the negative control, and lanes 7-9 are positive controls using the isolated plasmids as templates for PCR.

**Table S1** List of plasmids used in this study.

| **Plasmid name** | **Description** | **Source** |
| --- | --- | --- |
| pSL2740 | The plasmid for strength assay of promoter PR*_cpcB_*, driving gene *eyfp* | This study |
| pSL2741 | The plasmid for strength assay of promoter PR*_rbcL_*, driving gene *eyfp* | This study |
| pSL2744 | The plasmid for strength assay of promoter PR_sll1321_, driving gene *eyfp* | This study |
| pSL2745 | The plasmid for strength assay of promoter PR_sll1626_, driving gene *eyfp* | This study |
| pSL2746 | The plasmid for strength assay of promoter PR_sll5036_, driving gene *eyfp* | This study |
| pSL2748 | The plasmid for strength assay of promoter PR_sll1268_, driving gene *eyfp* | This study |
| pSL2749 | The plasmid for strength assay of promoter PR_sll1514_, driving gene *eyfp* | This study |
| pSL2750 | The plasmid for strength assay of promoter PR_slr0701_, driving gene *eyfp* | This study |
| pSL2751 | The plasmid for strength assay of promoter PR_ssl0452_, driving gene *eyfp* | This study |
| pSL2752 | The plasmid for strength assay of promoter PR_ssl2501_, driving gene *eyfp* | This study |
| pSL2753 | The plasmid for strength assay of promoter PR_ssr2227_, driving gene *eyfp* | This study |
| pSL2810 | pPMQAK1 with RSF1010 origin of replication | Huang et al.^*^ |
| pSL3020 | The shuttle vector composed by fragments from plasmids pCB2.4 and pSC101 | This study |
| pSL3104 | The plasmid for strength assay of promoter PR*_psbA2_*, driving gene *eyfp* | This study |
| pSL3105 | The plasmid for strength assay of promoter PR*_trc1O_*, driving gene *eyfp* | This study |
| pSL3106 | The plasmid for strength assay of RBS-*cpcB*, followed by gene *eyfp* | This study |
| pSL3107 | The plasmid for strength assay of RBS-*rbcL*, followed by gene *eyfp* | This study |
| pSL3108 | The plasmid for strength assay of RBS-*psbD*, followed by gene *eyfp* | This study |
| pSL3109 | The plasmid for strength assay of RBS-*psbA2*, followed by gene *eyfp* | This study |
| pSL3110 | The plasmid for strength assay of RBS-*psaA*, followed by gene *eyfp* | This study |
| pSL3111 | The plasmid for strength assay of RBS-*apcA*, followed by gene *eyfp* | This study |
| pSL3112 | The plasmid for strength assay of RBS-*psbB*, followed by gene *eyfp* | This study |
| pSL3113 | The plasmid for strength assay of RBS-*petH*, followed by gene *eyfp* | This study |
| pSL3114 | The plasmid for strength assay of RBS-*petB*, followed by gene *eyfp* | This study |
| pSL3115 | The plasmid for strength assay of RBS-*psbE*, followed by gene *eyfp* | This study |
| pSL3116 | The plasmid for strength assay of RBS-*psaC*, followed by gene *eyfp* | This study |
| pSL3117 | The plasmid for strength assay of RBS-*psaF*, followed by gene *eyfp* | This study |
| pSL3118 | The plasmid for strength assay of RBS-*psaL*, followed by gene *eyfp* | This study |
| pSL3119 | The plasmid for strength assay of RBS-*petF*, followed by gene *eyfp* | This study |
| pSL3120 | The plasmid for strength assay of RBS-*atpC*, followed by gene *eyfp* | This study |
| pSL3121 | The plasmid for strength assay of RBS-*ndhC*, followed by gene *eyfp* | This study |
| pSL3122 | The plasmid for strength assay of RBS-*ndhJ*, followed by gene *eyfp* | This study |
| pSL3123 | The plasmid for strength assay of RBS-*ndhG*, followed by gene *eyfp* | This study |
| pSL3124 | The plasmid for strength assay of RBS-*cydA*, followed by gene *eyfp* | This study |
| pSL3125 | The plasmid for strength assay of RBS-*gap2*, followed by gene *eyfp* | This study |
| pSL3126 | Containing blue fluorescence protein coding gene *bfp* driven by P*_trc1O_* with RBS-*cpcB* | This study |
| pSL3127 | The plasmid for strength assay of terminator T*_rrnB_* | This study |
| pSL3128 | The plasmid for strength assay of terminator T*_psbO_* | This study |
| pSL3129 | The plasmid for strength assay of terminator T*_rbcS_* | This study |
| pSL3130 | The plasmid for strength assay of terminator T*_atpC_* | This study |
| pSL3131 | The plasmid for strength assay of terminator T*_psbC_* | This study |
| pSL3132 | The plasmid for strength assay of terminator T*_psaB_* | This study |
| pSL3133 | The plasmid for strength assay of terminator T*_apcC_* | This study |
| pSL3134 | The plasmid for strength assay of terminator T*_psaC_* | This study |
| pSL3135 | The shuttle vector composed by fragments from plasmids pCA2.4 and pUC118 | This study |
| pSL3136 | pSL3020 containing gene *eyfp* driven by PR*_trc1O_* with terminator T*_rrnB_* | This study |
| pSL3137 | pSL3135 containing gene *eyfp* driven by PR*_trc1O_* with terminator T*_rrnB_* | This study |

^*^Huang H-H, Camsund D, Lindblad P, Heidorn T. Design and characterization of molecular tools for a synthetic biology approach towards developing cyanobacterial biotechnology. Nucleic Acids Res. 2010; 38:2577–93.

**Table S2** Sequences of promoters and terminators used in this study.

| **Element name** | **Sequence** |
| --- | --- |
| **PR*_cpcB_***  (corresponding gene function: Phycocyanin beta subunit.) | ATCTATACCCACCTGTAGAGAAGAGTCCCTGAATATCAAAATGGTGGGATAAAAAGCTCAAAAAGGAAAGTAGGCTGTGGTTCCCTAGGCAACAGTCTTCCCTACCCCACTGGAAACTAAAAAAACGAGAAAAGTTCGCACCGAACATCAATTGCATAATTTTAGCCCTAAAACATAAGCTGAACGAAACTGGTTGTCTTCCCTTCCCAATCCAGGACAATCTGAGAATCCCCTGCAACATTACTTAACAAAAAAGCAGGAATAAAATTAACAAGATGTAACAGACATAAGTCCCATCACCGTTGTATAAAGTTAACTGTGGGATTGCAAAAGCATTCAAGCCTAGGCGCTGAGCTGTTTGAGCATCCCGGTGGCCCTTGTCGCTGCCTCCGTGTTTCTCCCTGGATTTATTTAGGTAATATCTCTCATAAATCCCCGGGTAGTTAACGAAAGTTAATGGAGATCAGTAACAATAACTCTAGGGTCATTACTTTGGACTCCCTCAGTTTATCCGGGGGAATTGTGTTTAAGAAAATCCCAACTCATAAAGTCAAGTAGGAGATTAATTCASTART CODON |
| **PR*_trc1O_*** | GACGTCTAAGAAACCATTATTATCATGACATTAACCTATAAAAATAGGCGTATCACGAGGCAGAATTTCAGATAAAAAAAATCCTTAGCTTTCGCTAAGGATGATTTCTGGAATTCGCGGCCGCATCTAGAGTTGACAATTAATCATCCGGCTCGTATAATGTGTGGAATTGTGAGCGGATAACAATTTCACACATACTAGAAAAGAGGAGAAATACTAGSTART CODON |
| **PR*_rbcL_***  (corresponding gene function: ribulose bisphosphate carboxylase large subunit.) | AATTACTTCCCCCGCCGATGTGCAATACCAAGCCGCCTTAGATTTGCTCACCGGAGGAGTGGCAATCGCCCATAAATCTTCTTCAATTCCCGCCATGGCAACGGCTCACAAGCCCAACTAATCACCATTTGGACAAAACATCAGGAATTCTAATTAGAAAGTCCAAAAATTGTAATTTAAAAAACAGTCAATGGAGAGCATTGCCATAAGTAAAGGCATCCCCTGCGTGATAAGATTACCTTCAGAAAACAGATAGTTGCTGGGTTATCGCAGATTTTTCTCGCAACCAAATAACTGTAAATAATAACTGTCTCTGGGGCGACGGTAGGCTTTATATTGCCAAATTTCGCCCGTGGGAGAAAGCTAGGCTATTCAATGTTTATGGAGGACTGACCTAGSTART CODON |
| **PR*_psbA2_***  (corresponding gene function: photosystem II D1 protein.) | CTCCATTGTCCCTGAAAATCAGTTGTGTCGCCCCTCTACACAGCCCAGAACTATGGTAAAGGCGCACGAAAAACCGCCAGGTAAACTCTTCTCAACCCCCAAAACGCCCTCTGTTTACCCATGGAAAAAACGACAATTACAAGAAAGTAAAACTTATGTCATCTATAAGCTTCGTGTATATTAACTTCCTGTTACAAAGCTTTACAAAACTCTCATTAATCCTTTAGACTAAGTTTAGTCAGTTCCAATCTGAACATCGACAAATACATAAGGAATTATAACCAASTART CODON |
| **PR_sll1626_**  (corresponding gene function: SOS function regulatory protein.) | TCTTCGGCGATAACAGTAACATTTTTCGGGGTAGTCTCAATTTTGCTTTTTCGCCCTCAATCACAGCGGCGATCGCCTTCTGCTGGCCGAAAATGAGGAACCGTCGGAAGAAATGTAAAATTTTGTATCTTTTTTAGTATGATTGCCCTGAATGTTATGACTGGGTTTAAAATAGTCCTAGAGTCCTAAATACATTCCTATAGGAGATATTACSTART CODON |
| **PR_sll1514_**  (corresponding gene function: 16.6 kDa small heat shock protein, molecular chaperone.) | GTAAAGGTCTCCATTCCTATGGTTGGTTTTTATAGTTTTTCACAACCTAAGACTTCCTTCCAAAAATCCATAGGGCGGTGGAAGCTTAGCTATTTTTACCATTTTGTTTTGCCACTCAAATATTTACTTAAGGTGAGGTAAAAACTCATCTTTTTTTTACTAAAAATTGCGGCTAGAAATGTAATTTCGGCAATCCCCCCACCTTCTTTCCTGAAAACCGAATCTAACCTGGAAGGGGAAATTTTAAGATAGAACCATTCAAGGGTAATCAATTCCTTCCACACATCAGGAGTTAACATTSTART CODON |
| **PR_sll5036_**  (corresponding gene function: sulfide-quinone reductase.) | GAGTTTTTGTTGGAATGCCTTCTGTAGATTCATTTCTGTCATTATTCCCATGCCTTCTTTTGCTAGGGAATCCTTTACTAATCCGATAGCTTCATCAAATCCTACCAGCAATTTTTTACTAAAGTAATACATTTTATATCTCTGATTTTTATTGAACTAGTCCTTGCCAAATCGAACAAGCACCAATATTATAAGAATATAACTACATAGTTGTATTCGTCAATAGTTTTTGGGGGGAGGGAGTTTAAASTART CODON |
| **PR_ssl0452_**  (corresponding gene function: phycobilisome degradation protein,NblA.) | CAATGACCCAATAACTCGTACTGTTATCTACGTGGTGAAAGCCAAAAAGACGAACAGTTTAGCCTCCTCCTCCTCGGCGATCGCCAAGCGAAATGTCATGGGAGATGTTCAGATTGAGCATTTTTTTCTAAAAGCCCTTGCTAAAACAAACCACATGTGCAGGGTGTCCCCGATGTTGACTAAATTCAGCGGACTTAAAACCTATTTTTTCCCTGGGTTGCTAGGTTTGCCCCCCGTTTTGGGCAAGCTTGTATAAGCAGATACTGTTAATTGGGTCAACTTTTTGTTACATTTATTTACAATTGATTGTTTACAATTGAAAGGTAGTCGCCTTGGAGGGCAACAGCTSTART CODON |
| **PR_ssl2501_**  (corresponding gene function: unknown protein.) | CAGAAAACTAATCTGCCCTTTGCCATCAATTTCTAATCACCGTAGTGAACAGATTGTCCATGATAACCACTGGCGATCGCCGCTTCTGCTTCTAAGCTCAGCTTTTAGGGTTGACTAGTGAAAAGAAGGGGGCAAAAGATGTCAGACTTTGGTGATATTCCCCCGGACTTTTCCCTGGGGAGGCCAAGGCCAATCACTATAATGGAGACGTGCGATACCGATATTCATTGGAAGTGGTCTGCACCGCCCTGCTTCCCCTTAAACCCTTGAACGTTTAGGGATATTAGCGGTTTTAGCCCTSTART CODON |
| **PR_sll1268_**  (corresponding gene function: unknown protein.) | AGCTAACCCCATTTGATTAGCTAATTTTTACCCTTCTTTCCCCTGGCGATCGCCTTTGGCCCTGCCTTTTCAGGCTATAGAAGTGGTCTGGGTCACCCAATAGTTGTGATCTAGATCACAGAGGGCCACGGCCTGGATCTCTACAATGGTCAATGGGCAGTATGCCTAATCTGCCTGTTTGGGGCCATGCAAAGGACATAGTTTTCTATGGTTAGTCCAGGTTTGACGCCTCTGCCAAAGTCCCTCCACCTGCTGGTTGCTTTTAASTART CODON |
| **PR_sll1321_**  (corresponding gene function: unknown protein.) | GGAGAATTGGGGGGAAGAACCATAGCCGCTGGGAAAAAACGCTGGACGGGATAGGGGCAACTGGTAGGCCTTGGATAATGATCAGAATCAGCCACCTAGGTGATCTTGGCAATTTTCTTTGGCGATCGCCAGGGGGATTGCCGTCCTTGTTGGGGGTCTAGGCAAAAAACAAAAAACTAAGAATCCGTTGCGACAGCGTGATATGATCGCTTCTGGCTTAGGGTAGGTGGTCCTTGATTACCAAGGAAGACCGTTGCCCAAACTCCATTTCTCACCTTGCTAGTAGTCAGAGTSTART CODON |
| **PR_ssr2227_**  (corresponding gene function: transposase.) | AGTGAATAAAGTTAAATATTGCTTGCCATTGTTGGTGGGATTTTGTACATTGAATACATGGAGCCGAAGGGCTTCACTGGCAGATAAAAACAGCCACTTCCGTCCGAAAACCCTAGTTTAACAAGAGAGGTAAAACCGAGCTGTTTCTTTCTAAATCTGTCTCGTTTGTTTCTCAGTTCATCGCTCAGATGACGGACAACACCCAAACCGAAAATAGTCGCAACCCCAATTGCTAGCCATGGGAGATAGGCACTGACCTTGTATTAGGTATTTGCAGCTCCGGTCATGCTCGAAGAAAGGAGGGTAAGTCGAACTAGTTACTAGGTTCTGGGTGTTGTTTGGAAGTGATTTCTAGGGAAAATATAAAGAAAAGAAAATATAAAGAAAAGAGGATAGGAGAAATCTATTTTTTATGGTGAGAGGATAAAAGTTGAGTATTGTCGATGAGTAGTTCGCCGAAGGTATTGAATCCTCAGGGCAAGCCATATGCTAGTGACACGAGTGATGAGGAGTGGAAGGTAATTGAGCCCAAGTCACCAGCCCAGCACCAAAAGGATTTGGGCATCCTAGAGAGTTAGACCTACGAGAAATAAATTATCASTART CODON |
| **PR_slr0701_**  (corresponding gene function: predicted regulatory protein.) | CGAGAACTAAGACAAAAATTACTGGGGGTTAGGTAGGCGATCGCCAAATTACGAATATTGCTCCGAAACTGACCACATTACCCACTGCGAAGAGTTCTAGCTATCCTTTACTGATAAGAAACTGGGAGAAAATCGTCCTTTTTTTGTAACAAACTATATCAACCTACAACCTTATACCTAAGTATAGGGAATATATTGTAGTAGAGACAGAGAAGAAATGGGAGGSTART CODON |
| **T*_rrnB_*** | STOP CODONTACTAGAGCCAGGCATCAAATAAAACGAAAGGCTCAGTCGAAAGACTGGGCCT TTCGTTTTATCTGTTGTTTGTCGGTGAACGCTCTCTACTAGAGTCACACTGGCTCACCTTCGGGTGGGCCTTTCTGCGTTTATATACTAGTAGCGGCCGCTGCAGTCCGGCAAAAAAACGGGCAAGGTGTCACCACCCTGCCCTTTTTCTTTAAAACC |
| **T*_atpC_*** | STOP CODONCCCATTTAGTGATCTTTTCGTTGCACTCCTGCTTGAACTAGCGGGAGTTTTTTACT TCAGGGCCTTCATGCCTCTCTAACGCTTCGGACATACTGATATTAAGATTCAGGTGACAATTCTTTGTTGAGATTTGACCAAGCGCCGATCGCC |
| **T*_rbcS_*** | STOP CODONTTACAGTTTTGGCAATTACTAAAAAACTGACTTCAATTCAATGTTAGCCCGCTCC CGCGGGTTTTTTGTTGCTTTTTCACAGTGACTATAGGTAATCAGCAACACAATACGGCCCTGTTCTTTGGACAGTTTTTGTATAATGTTGACCGCATCCTGACCGGATTTTTTATCTAAGTGGGGAATTGTCAATTGTCAATTA |
| **T*_psbO_*** | STOP CODONTTTTTTGTTGTGGTAGCTGACTGGCTTTTTAGTTAGTTACTTTGACGAGCTGAAC AGTTTGGCGATCGCCGTTAAGGTGGTCGTCTTTTTCTATCAAATTAATTCAACTAAGGTATTGATTCTTTTAAGTATTTTCTTGTCGTTTTGTTG |
| **T*_psaC_*** | STOP CODONATCGTATAGATCTGGTAACTGGCCCCGCCATTATTCCAATTTTTGATCAAACTTT GACAGCTTTCGAGCTGGGAAGGCCCCTGGGGTCTTCTTTTTTATTTGTTTGTTTGTCAACATTCAATCTAAGTTTGTTCACCTGCACGAAATAATCCGAAAGTTAATGTTTTTACCAGAGCGGCGATTTTCGACCAAAGC |
| **T*_psaB_*** | STOP CODONCGAATTCCTCTGTTAGGTAATTAAGCTTGTCCCCTGCCCTCGTTGGTGGGGGATT TGCTTTAATTGGCTGATCGCCACAAAAAATGGATTGGAATCGGCCTCATTTAATCTTAAGTTTTTTGTTATTTTTACCCCATTGC |
| **T*_apcC_*** | STOP CODONGCTTGAGACTAGTTGTTGCTAATTTAGCAATGTTGAATGGCAATTTTATGAATCA ATTTTTTCGAGGTCAGCAATGGCCTCTTTTTTTGTTTGGTTAATCTCCCTCTTCCCTTCTGGCCTGAAAGCAACACCATTCCTGCCGTTTCCATATAGCGACCACCGTCCAACCGTTTTGCTCCAGCGCATCGGCGATCGCCTGA |
| **T*_psbC_*** | STOP CODONATTGAGACTTTTCTGATTTTGCAAAGGTTTTGCTTTAGTTAAACCCAATTGATTA GTGTCCCCTGCCCATTTGGTGGGGGATTATTATTTTTAAGATAATCCTATTTTTTGGAGTGAGGCCAGTTACCTATTAGACGCGCGACTCGAAAGTCGTTCAGGGGAGTTGGAACGGCTTCCAAAAACCTTTCCCCGCTGGTGTT |

The 22-bp underline sequence in P*_trc1O_* was used to switch various sequences for RBS assay.

The sequences in green font in terminators are GC-rich hairpin structures predicted by the tool ARNold^*^

^*^Naville M, Ghuillot-Gaudeffroy A, Marchais A, Gautheret D. ARNold: a web tool for the prediction of Rho-independent transcription terminators. RNA Biol. 2011; 8:11-3.

**Table S3** List of primers used in this study.

| **Primer name** | **Sequence 5' -> 3'** | **Purpose of primer** |
| --- | --- | --- |
| Testing_PcpcB_F | GCTTTCCTGGCTTTGCTTCCATCTATACCCACCTGTAGAGAAG | For fragment P*_cpcB_* |
| Testing_PcpcB_R | TCCTCGCCCTTGCTCACCATTGAATTAATCTCCTACTTGAC |  |
| Testing_PrbcL_F | GCTTTCCTGGCTTTGCTTCCAATTACTTCCCCCGCCGATG | For fragment P*_rbcL_* |
| Testing_PrbcL_R | TCCTCGCCCTTGCTCACCATCTAGGTCAGTCCTCCATAAAC |  |
| Testing_Psll1321_F | GCTTTCCTGGCTTTGCTTCCGGAGAATTGGGGGGAAGAAC | For fragment P_sll1321_ |
| Testing_Psll1321_R | TCCTCGCCCTTGCTCACCATACTCTGACTACTAGCAAGGTG |  |
| Testing_Psll1626_F | GCTTTCCTGGCTTTGCTTCCTCTTCGGCGATAACAGTAAC | For fragment P_sll1626_ |
| Testing_Psll1626_R | TCCTCGCCCTTGCTCACCATGTAATATCTCCTATAGGAATG |  |
| Testing_Psll5036_F | GCTTTCCTGGCTTTGCTTCCGGAGTTTTTGTTGGAATGCC | For fragment P_sll5036_ |
| Testing_Psll5036_R | TCCTCGCCCTTGCTCACCATTTTAAACTCCCTCCCCCCAAAAAC |  |
| Testing_Psll1268_F | GCTTTCCTGGCTTTGCTTCCAGCTAACCCCATTTGATTAG | For fragment P_sll1268_ |
| Testing_Psll1268_R | TCCTCGCCCTTGCTCACCATTTAAAAGCAACCAGCAGGTG |  |
| Testing_Psll1514_F | GCTTTCCTGGCTTTGCTTCCGTAAAGGTCTCCATTCCTATG | For fragment P_sll1514_ |
| Testing_Psll1514_R | TCCTCGCCCTTGCTCACCATAATGTTAACTCCTGATGTGTG |  |
| Testing_Pslr0701_F | GCTTTCCTGGCTTTGCTTCCCGAGAACTAAGACAAAAATTAC | For fragment P_slr0701_ |
| Testing_Pslr0701_R | TCCTCGCCCTTGCTCACCATCCTCCCATTTCTTCTCTGTC |  |
| Testing_Pssl0452_F | GCTTTCCTGGCTTTGCTTCCCAATGACCCAATAACTCGTAC | For fragment P_ssl0452_ |
| Testing_Pssl0452_R | TCCTCGCCCTTGCTCACCATAGCTGTTGCCCTCCAAGGCG |  |
| Testing_Pssl2501_F | GCTTTCCTGGCTTTGCTTCCCAGAAAACTAATCTGCCCTTTG | For fragment P_ssl2501_ |
| Testing_Pssl2501_R | TCCTCGCCCTTGCTCACCATAGGGCTAAAACCGCTAATATC |  |
| Testing_Pssr2227_F | GCTTTCCTGGCTTTGCTTCCAGTGAATAAAGTTAAATATTG | For fragment P_ssr2227_ |
| Testing_Pssr2227_R | TCCTCGCCCTTGCTCACCATTGATAATTTATTTCTCGTAG |  |
| Testing_PpsbA2_F | TTCCTGGCTTTGCTTCCCACCTCCATTGTCCCTGAAAATCAG | For fragment P*_psbA2_* |
| Testing_PpsbA2_R | TCCTCGCCCTTGCTCACCATTTGGTTATAATTCCTTATGTATTTG |  |
| Testing_Ptrc1O_F | TTCCTGGCTTTGCTTCCCACGACGTCTAAGAAACCATTATTATC | For fragment P*_trc1O_* |
| Testing_Ptrc1O_R | TCCTCGCCCTTGCTCACCATCTAGTATTTCTCCTCTTTTCTAGTATG |  |
| Testing_EYFP_F | ATGGTGAGCAAGGGCGAGGAG | For fragment of gene *eyfp* and terminator T*_rrnB_* |
| Testing_TrrnB_R | TACGCTGACTTGACGGGACACGGTTTTAAAGAAAAAGGGCAG |  |
| Testing_EYFP_F | ATGGTGAGCAAGGGCGAGGAG |  |
| Testing_EYFP_R1 | ACGGGAGGTTTTAAAGAAAAAGGGCAGG |  |
| Testing_EYFP_R2 | AGAGCATTACGCTGACTTGACGGGAGGTTTTAAAGAAAAAG |  |
| BkRSF_F1 | TCCCGTCAAGTCAGCGTAATGC | For plasmid pSL2810 |
| BkRSF_R2 | GGAAGCAAAGCCAGGAAAGCG |  |
| BkRSF_middle_F2 | ACTTACAGGAATACCGGGAGGC |  |
| BkRSF_middle_R1 | GGTCTATTGCCTCCCGGTATTC |  |
| RBStest_Ptrc_F | AGCTCCATAGGCCGCTTTCC | For fragment of P*_trc1O_* and T*_rrnB_* |
| RBStest_TrrnB_R | CTGGCAGAGCATTACGCTGAC |  |
| RBStest_Ptrc_rbscpcB_R | TGAATTAATCTCCTACTTGACTGTATGTGTGAAATTGTTATCCGCTC | For assay of RBS-*cpcB* |
| RBStest_EYFP_rbscpcB_F | AGTCAAGTAGGAGATTAATTCAATGGTGAGCAAGGGCGAGGAG |  |
| RBStest_Ptrc_rbsrbcL_R | CTAGGTCAGTCCTCCATAAACAGTATGTGTGAAATTGTTATCCGCTC | For assay of RBS-*rbcL* |
| RBStest_EYFP_rbsrbcL_F | TGTTTATGGAGGACTGACCTAGATGGTGAGCAAGGGCGAGGAG |  |
| RBStest_Ptrc_rbspsbD_R | AAATGCAAATCCTCTTGCGTAGGTATGTGTGAAATTGTTATCCGCTC | For assay of RBS-*psbD* |
| RBStest_EYFP_rbspsbD_F | CTACGCAAGAGGATTTGCATTTATGGTGAGCAAGGGCGAGGAG |  |
| RBStest_Ptrc_rbspsbA2_R | TTGGTTATAATTCCTTATGTATGTATGTGTGAAATTGTTATCCGCTC | For assay of RBS-*psbA2* |
| RBStest_EYFP_rbspsbA2_F | ATACATAAGGAATTATAACCAAATGGTGAGCAAGGGCGAGGAG |  |
| RBStest_Ptrc_rbspsaA_R | GCAGGGTTCTCCTCGCTCGACAGTATGTGTGAAATTGTTATCCGCTC | For assay of RBS-*psaA* |
| RBStest_EYFP_rbspsaA_F | TGTCGAGCGAGGAGAACCCTGCATGGTGAGCAAGGGCGAGGAG |  |
| RBStest_Ptrc_rbsapcA_R | GGATGGATTCCTCCGTAAAGATGTATGTGTGAAATTGTTATCCGCTC | For assay of RBS-*apcA* |
| RBStest_EYFP_rbsapcA_F | ATCTTTACGGAGGAATCCATCCATGGTGAGCAAGGGCGAGGAG |  |
| RBStest_Ptrc_rbspsbB_R | TGACGCTCCTTCTAGTAACGAAGTATGTGTGAAATTGTTATCCGCTC | For assay of RBS-*psbB* |
| RBStest_EYFP_rbspsbB_F | TTCGTTACTAGAAGGAGCGTCAATGGTGAGCAAGGGCGAGGAG |  |
| RBStest_Ptrc_rbspetH_R | GGGTTAATTGCTCCCTACTCAAGTATGTGTGAAATTGTTATCCGCTC | For assay of RBS-*petH* |
| RBStest_EYFP_rbspetH_F | TTGAGTAGGGAGCAATTAACCCATGGTGAGCAAGGGCGAGGAG |  |
| RBStest_Ptrc_rbspetB_R | TGAAGCTAGACTTCTCTTAAGGGTATGTGTGAAATTGTTATCCGCTC | For assay of RBS-*petB* |
| RBStest_EYFP_rbspetB_F | CCTTAAGAGAAGTCTAGCTTCAATGGTGAGCAAGGGCGAGGAG |  |
| RBStest_Ptrc_rbspsbE_R | TTTGTGTTCTCCTTCAATTTTTGTATGTGTGAAATTGTTATCCGCTC | For assay of RBS-*psbE* |
| RBStest_EYFP_rbspsbE_F | AAAAATTGAAGGAGAACACAAAATGGTGAGCAAGGGCGAGGAG |  |
| RBStest_Ptrc_rbspsaC_R | TGACTATCGGCTCCTTAACCGAGTATGTGTGAAATTGTTATCCGCTC | For assay of RBS-*psaC* |
| RBStest_EYFP_rbspsaC_F | TCGGTTAAGGAGCCGATAGTCAATGGTGAGCAAGGGCGAGGAG |  |
| RBStest_Ptrc_rbspsaF_R | AAGAATCGTTTCCTTGGTTAAAGTATGTGTGAAATTGTTATCCGCTC | For assay of RBS-*psaF* |
| RBStest_EYFP_rbspsaF_F | TTTAACCAAGGAAACGATTCTTATGGTGAGCAAGGGCGAGGAG |  |
| RBStest_Ptrc_rbspsaL_R | TGGTATTGAGTTCTCCTATTAAGTATGTGTGAAATTGTTATCCGCTC | For assay of RBS-*psaL* |
| RBStest_EYFP_rbspsaL_F | TTAATAGGAGAACTCAATACCAATGGTGAGCAAGGGCGAGGAG |  |
| RBStest_Ptrc_rbspetF_R | AGATGTTTCCTCTTCTCAAAAAGTATGTGTGAAATTGTTATCCGCTC | For assay of RBS-*petF* |
| RBStest_EYFP_rbspetF_F | TTTTTGAGAAGAGGAAACATCTATGGTGAGCAAGGGCGAGGAG |  |
| RBStest_Ptrc_rbsatpC_R | TTTTAGGAATTTCCCTTTGATAGTATGTGTGAAATTGTTATCCGCTC | For assay of RBS-*atpC* |
| RBStest_EYFP_rbsatpC_F | TATCAAAGGGAAATTCCTAAAAATGGTGAGCAAGGGCGAGGAG |  |
| RBStest_Ptrc_rbsndhC_R | AGGGCCAATGGTCTCCTCACGGGTATGTGTGAAATTGTTATCCGCTC | For assay of RBS-*ndhC* |
| RBStest_EYFP_rbsndhC_F | CCGTGAGGAGACCATTGGCCCTATGGTGAGCAAGGGCGAGGAG |  |
| RBStest_Ptrc_rbsndhJ_R | GGTTTAATTGCTCCTTTTGTTGGTATGTGTGAAATTGTTATCCGCTC | For assay of RBS-*ndhJ* |
| RBStest_EYFP_rbsndhJ_F | CAACAAAAGGAGCAATTAAACCATGGTGAGCAAGGGCGAGGAG |  |
| RBStest_Ptrc_rbsndhG_R | AGTGGTTAATCTCCTAGGTACGGTATGTGTGAAATTGTTATCCGCTC | For assay of RBS-*ndhG* |
| RBStest_EYFP_rbsndhG_F | CGTACCTAGGAGATTAACCACTATGGTGAGCAAGGGCGAGGAG |  |
| RBStest_Ptrc_rbscydA_R | CTGTAAAGAGCGCTCCTAAATGGTATGTGTGAAATTGTTATCCGCTC | For assay of RBS-*cydA* |
| RBStest_EYFP_rbscydA_F | CATTTAGGAGCGCTCTTTACAGATGGTGAGCAAGGGCGAGGAG |  |
| RBStest_Ptrc_rbsgap2_R | GTTCGTCTTGCCCTCTCTGTTTGTATGTGTGAAATTGTTATCCGCTC | For assay of RBS-*gap2* |
| RBStest_EYFP_rbsgap2_F | AAACAGAGAGGGCAAGACGAACATGGTGAGCAAGGGCGAGGAG |  |
| Ter_mBFP_F | AGTCAAGTAGGAGATTAATTCAATGCAGAATCTGAACGGCAAAG | For fragment *bfp* |
| Ter_mBFP_R | TCAAGCGGCGAAGCCGCCGTC |  |
| Ter_TrrnB_F | GGACGGCGGCTTCGCCGCTTGATACTAGAGCCAGGCATCAAATAAAAC | For fragment T*_rrnB_* |
| Ter_TrrnB_R | TGAATTAATCTCCTACTTGACTGGTTTTAAAGAAAAAGGGCAGG |  |
| Ter_TrbcS_F1 | GGACGGCGGCTTCGCCGCTTGATTACAGTTTTGGCAATTACTAAAAAAC | For fragment T*_rbcS_* |
| Ter_TrbcS_R1 | TTACGCTGACTTGACGGGACACTAATTGACAATTGACAATTCCCCAC |  |
| Ter_TpsbO_F | GGACGAGCTGTACAAGTAATAATTTTTTGTTGTGGTAGCTGACTG | For fragment T*_psbO_* |
| Ter_TpsbO_R | TGAATTAATCTCCTACTTGACTCAACAAAACGACAAGAAAATAC |  |
| Ter_TrbcS_F2 | GGACGAGCTGTACAAGTAATAATTACAGTTTTGGCAATTACTAAAAAAC | For fragment T*_rbcS_* |
| Ter_TrbcS_R2 | TGAATTAATCTCCTACTTGACTTAATTGACAATTGACAATTCCCCAC |  |
| Ter_TatpC_F | GGACGAGCTGTACAAGTAATAACCCATTTAGTGATCTTTTCGTTGC | For fragment T*_atpC_* |
| Ter_TatpC_R | TGAATTAATCTCCTACTTGACTGGCGATCGGCGCTTGGTCAAATC |  |
| Ter_TpsbC_F | GGACGAGCTGTACAAGTAATAAATTGAGACTTTTCTGATTTTGCAAAG | For fragment T*_psbC_* |
| Ter_TpsbC_R | TGAATTAATCTCCTACTTGACTAACACCAGCGGGGAAAGGTTTTTG |  |
| Ter_TpsaB_F | GGACGAGCTGTACAAGTAATAACGAATTCCTCTGTTAGGTAATTAAG | For fragment T*_psaB_* |
| Ter_TpsaB_R | TGAATTAATCTCCTACTTGACTGCAATGGGGTAAAAATAACAAAAAAC |  |
| Ter_TapcC_F | GGACGAGCTGTACAAGTAATAAGCTTGAGACTAGTTGTTGCTAATTTAG | For fragment T*_apcC_* |
| Ter_TapcC_R | TGAATTAATCTCCTACTTGACTTCAGGCGATCGCCGATGCGCTGG |  |
| Ter_TpsaC_F | GGACGAGCTGTACAAGTAATAAATCGTATAGATCTGGTAACTGGCCCCG | For fragment T*_psaC_* |
| Ter_TpsaC_R | TGAATTAATCTCCTACTTGACTGCTTTGGTCGAAAATCGCCGCTC |  |
| BKtest_pCA_F | GGGTTTTTTTATGAGCTTAAATATTAAATTATTTAATATTTC | For plasmid pSL3135 |
| BKtest_pCA_R | TTTTGCTGGCCTTTAATAGACTTACTCAATTAAGAAAATAGC |  |
| BKtest_pUC118_F | AGTAAGTCTATTAAAGGCCAGCAAAAGGCCAGG |  |
| BKtest_pUC118_R | GCGGTTTTTCCACGTGAACCATAGTACGCGCCCTGTAG |  |
| BKtest_Cm_F | TACTATGGTTCACGTGGAAAAACCGCCAGGTAAACTC | For fragment *Cm^R^* |
| BKtest_Cm_R | TAATATTTAAGCTCATAAAAAAACCCGCCGAAGCG |  |
| BKtest_PtrcEYFP_F1 | ACTTGGTCTGACAGTTACACGACGTCTAAGAAACCATTATTATC | For plasmid pSL3136 |
| BKtest_PtrcEYFP_R1 | CTGAGTTGAAGGATCAGCACGGTTTTAAAGAAAAAGGGCAGG |  |
| BKtest_PtrcEYFP_F2 | GGGCGCGTACTATGGTTCACGACGTCTAAGAAACCATTATTATC | For plasmid pSL3137 |
| BKtest_PtrcEYFP_R2 | TTACCTGGCGGTTTTTCCACGGTTTTAAAGAAAAAGGGCAGG |  |
| RSF1010_mid_F | AGCTCCATAGGCCGCTTTCC | Validation for pRSF1010 |
| RSF1010_mid_R | CTGGCAGAGCATTACGCTGAC |  |
| BkpUC118_mid_F | GGTGCCGTAAAGCACTAAATC | Validation for pCA-UC118 |
| OripCA2.4_mid_R | CCCTTTGCTTACTCATTTCAGAC |  |
| BkpSC101_mid_F | CTGTCCCTAGTGCTTGGATTC | Validation for pCB-SC101 |
| BkpCB2.4_mid_R | CACCTGGAAAATATAACACCTC |  |
